# Survey of haemosporidian parasite infections in an endangered high alpine bird

**DOI:** 10.1186/s13071-023-05667-7

**Published:** 2023-02-14

**Authors:** Angela N. Theodosopoulos, Garth M. Spellman, Scott A. Taylor

**Affiliations:** 1 Department of Ecology and Evolutionary Biology, University of Colorado at Boulder, Campus, Box 334, Boulder, CO USA; 2 Department of Zoology, Denver Museum of Nature and Science, 2001 N Colorado Blvd, Denver, CO USA

**Keywords:** Alpine specialist, Brown-capped Rosy-Finch, *Haemoproteus*, *Leucocytozoon*, Vector

## Abstract

The Brown-capped Rosy-Finch is an endangered high alpine specialist that breeds on cliffs in the Rocky Mountains of North America. We know little about the parasites that infect these birds but filling this knowledge gap is important for understanding their population decline. The aim of this study was to survey haemosporidian parasite infections in Brown-capped Rosy-Finches. We sampled 104 Brown-capped Rosy-Finches during their breeding season at six sampling sites spanning the Colorado Rocky Mountains where they are nearly endemic. We used nested PCR methods to screen birds for parasite infections, and Sanger sequencing data were used to identify parasite lineages. Four of the sampling sites had birds with infections. Females were more often infected than males (prevalence = 15.6% and 9.7%, respectively). We observed twice as many infected birds sampled in July compared to birds sampled in June (prevalence = 12.5% and 6.25%, respectively). The percent of infected birds by sampling site ranged from 0 to over 20%. In total we identified eight different genetic lineages of haemosporidian parasites infecting Brown-capped Rosy-Finches—seven were in the genus *Leucocytozoon*, and one was in the genus *Haemoproteus*. Network analysis clearly separates *Haemoproteus* from *Leucocytozoon*, with *Leucocytozoon* lineages comprising two major clusters. Based on reports made to the MalAvi database, all lineages in Cluster 1 have the same reported insect vector, *Simulium silvestre*. We report that Brown-capped Rosy-Finches experience infections with haemosporidian parasites and that the percentage of infected birds differed markedly between sampling sites. We hypothesize that vector ecology and associated variation in climate at sampling sites explain our observations.

**Supplementary Information:**

The online version contains supplementary material available at 10.1186/s13071-023-05667-7.

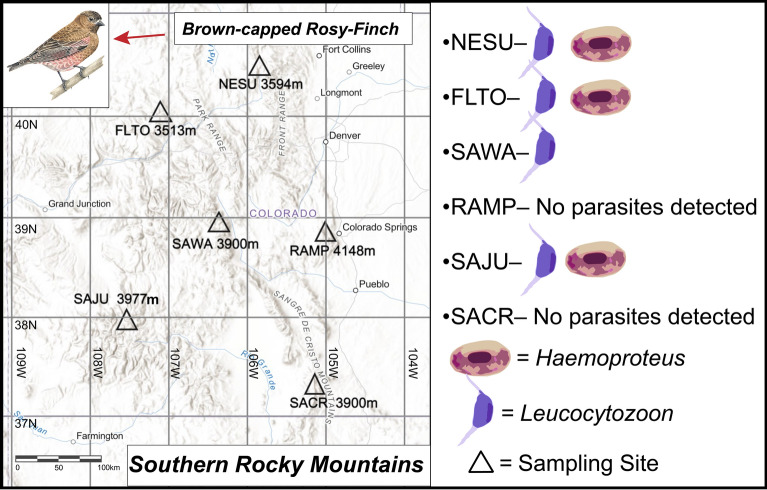

## Background

Host natural history and ecology play an essential role in shaping their parasite communities. Blood parasites transmitted by vectors include members of the order Haemosporida and, in birds, can involve the genera *Plasmodium*, *Leucocytozoon*, and *Haemoproteus* [1]. These parasites have complex life cycles and spend a portion of their life history infecting vertebrate host blood cells [1]. Haemosporidian parasites are widespread, diverse, and can influence host fitness [2–5]. For some avian hosts, such as long-distance migrants, exposure to blood-feeding insects and their associated haemosporidian parasites can occur at both wintering and breeding grounds [6, 7]. Conversely, for birds in temperate regions that are either non-migratory or are short-distance migrants, parasite transmission likely occurs during the breeding season, when insect vectors are active. Chronic infections can be retained across years [5], but for some haemosporidian parasites, infections are more commonly detected during the host breeding season [7, 8].

Brown-capped Rosy-Finches (*Leucosticte australis*) are Fringillidae songbirds and short-distance migrants that overwinter at lower elevations in the Southern Rocky Mountains where they are endemic [9]. During the summer, Brown-capped Rosy-Finches occur in extreme environments—they are among the highest altitudinal breeders in North America and nest on cliffs above treeline [9]. The Brown-capped Rosy-Finch is considered an endangered species whose population has declined by 95% since 1970 [10]. While climate-mediated habitat changes are suspected to be the primary cause of Brown-capped Rosy-Finch population decline, explicit investigations of other factors that might influence their conservation are nearly absent [11].

We know very little about the parasites that infect Brown-capped Rosy-Finches and the factors that shape their distributions and infection prevalence. To our knowledge only a single previous 1970 study investigated infections with vector-mediated blood parasites [12]. Given this existing knowledge gap, and the potential conservation implications, we sampled Brown-capped Rosy-Finches from throughout the Colorado Rocky Mountains for haemosporidian parasites. Sampling took place during the breeding season (June–July). Here we (i) report observed haemosporidian parasite prevalence by host sex, month, and site, (ii) identify the parasite lineages found, and (iii) report the occurrence of these lineages in other avian hosts as well as insect vectors using data from the MalAvi database [13].

## Methods

**Fig. 1:**
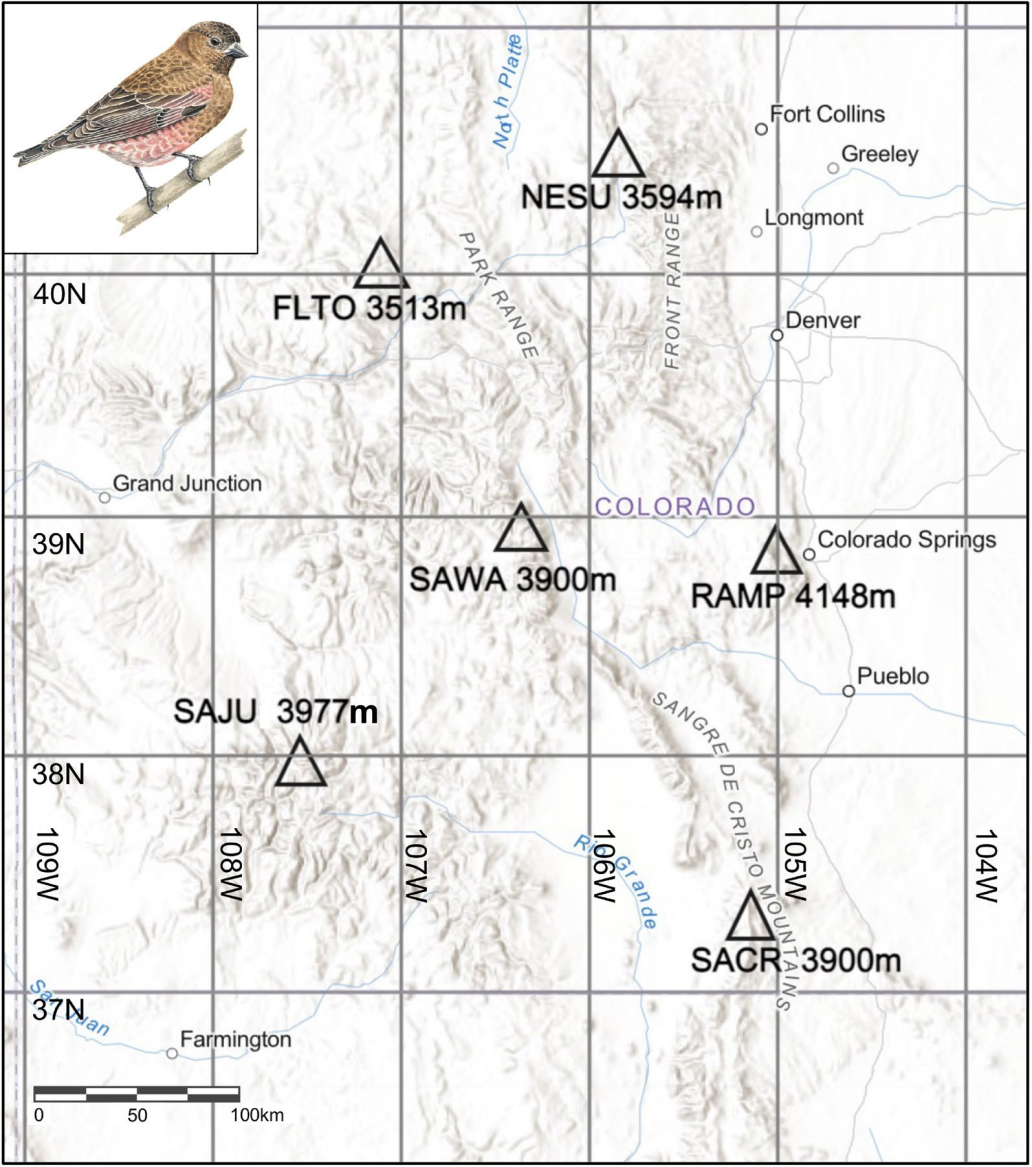
Sampling locations for Brown-capped Rosy-Finches (*Leucosticte australis*) are summarized by triangles with acronyms shown

During June and July 2018, we captured 104 Brown-capped Rosy-Finches from nine locations across six Colorado mountain ranges (Fig. 1, Table 1). We captured birds using Potter traps baited with black-oil sunflower seeds. We then banded each bird with a USGS metal band and collected blood from the brachial vein using a heparinized capillary tube and a 0.20-gauge needle prior to release. Blood samples were preserved in Queen’s lysis buffer. Capture sites were all above treeline and ranged in elevation from 3513 m (FLTO) up to 4148 m (RAMP). We conducted salt extractions to isolate and purify DNA from blood preserved in lysis buffer [14]. To screen birds for infection we used nested PCR reactions to amplify a region of the *cytb* gene, and all birds were screened at least twice [15]*.* Nested PCRs were conducted in 25-μl reactions. To screen birds for *Plasmodium* and *Haemoproteus*, we used the initial primers HAEMNF and HAEMNR2 with nested primers HAEMF and HAEMR2 [16]. We screened birds for *Leucocytozoon* lineages using initial primers DW2 and DW4, followed by nested primers LeucoF and LeucoR [17]. All PCRs included both positive and negative controls. We checked amplifications using gel electrophoresis with SYBR safe stain and fluorescence occurring at the expected length of the amplified product. We then submitted positive testing PCR samples for Sanger sequencing by QuintaraBio (Hayward, CA) to confirm infections and identify parasite lineages. We note that none of the birds were screened for infection using microscopy to examine blood smears.

**Table 1:** Colorado sites screened for haemosporidian parasites of Brown-capped Rosy-Finches (*Leucosticte australis*) in 2018

Site	Mountain range	Month sampled	Average sampling elevation (m)	# birds sampled	Proportion infected	SE of proportion infected
NESU	Never Summer	July	3594	23 (4 F, 19 M)	0.22	0.088
FLTO	Flat Tops	July	3513	18 (12 F, 6 M)	0.17	0.090
SAWA	Sawatch	June	3900	9 (4 F, 5 M)	0.11	0.111
RAMP	Rampart	June	4148	18 (3 F, 15 M)	0.00	0.000
SAJU	San Juan	June	3977	29 (6 F, 23 M)	0.10	0.058
SACR	Sangre De Cristo	July	3900	7 (3 F, 4 M)	0.00	0.000

We edited haemosporidian *cytb* sequences using Geneious prime v2020.1.2 [18]. First, we carried out a de novo assembly with the highest possible sensitivity for all sequences with forward and reverse primers for each bird in our study. We aligned the resulting sequence data to the MalAvi database [13] using the BLAST algorithm and identified parasite lineages. Birds were also screened for the presence of coinfections, which involved either infections with lineages in different genera (e.g. an infection with both *Leucocytozoon* and *Haemoproteus* parasites) or infections within the same genus (e.g. two different lineages in the genus *Leucocytozoon*). Some coinfections could be identified from Sanger sequencing data that resulted from the different sets of primers used. Other coinfections were identified based on the presence of mixed peaks that could be visualized in chromatograms. A mixed peak means a sequence might have two possible base pairs at the associated site. These coinfections were identified to lineage by duplicating the sequence and then assigning one of the possible base pairs to each sequence and re-aligning them to the MalAvi database. Importantly, all observed within-genus coinfections were lineages that only differed by a single base pair in their *cytb* sequences.

Prevalence is the proportion of infected individuals within a sampling population. We calculated prevalence partitioned by sampling site, sex, and month of sampling and estimated associated standard errors using R v4.1.2 [19]. To compare the genetic similarity of our observed haemosporidian lineages, and their distributions by sampling site, we conducted a network analysis using the program PopArt [20]. We applied the default settings for the Median-Joining method to calculate the network [21]. Importantly, while Median-Joining networks are not rooted, and therefore cannot show evolutionary directionality [22], they can be used to visualize reticulation events for closely related lineages [21].

## Results

**Fig. 2:**
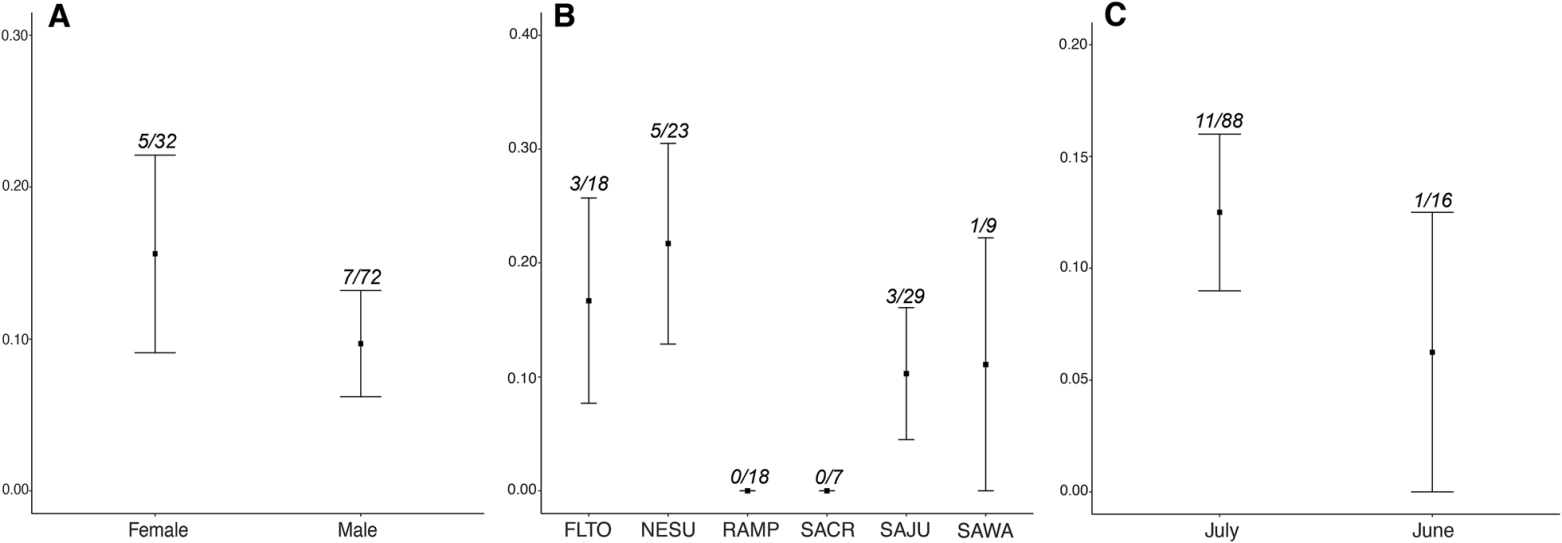
Proportion of infected birds partitioned by sex **A**, sampling site **B**, and sampling month **C** with associated standard errors. The number of infected birds in proportion to total birds sampled for each category is shown above error bars

Twelve of the 104 Brown-capped Rosy-Finches that we screened for haemosporidian parasites were infected (prevalence = 11.5%, Additional file 1: Dataset S1). We identified 10 birds with *Leucocytozoon* infections (prevalence = 9.6%) and five birds with *Haemoproteus* infections (prevalence = 4.8%). We reported information on lineages we found infecting Brown-capped Rosy-Finches to the MalAvi database [13]. We did not identify any lineages that had not previously been described; therefore, we did not upload these sequences to GenBank. No birds had detectable infections with *Plasmodium* parasites. Coinfections involving both *Leucocytozoon* and *Haemoproteus* lineages were detected in three birds (prevalence = 2.9%), and two birds had coinfections with two *Leucocytozoon* lineages (prevalence = 1.9%). We observed more infections in female Brown-capped Rosy-Finches than males (prevalence = 15.6%, 5/32 infected; 9.7%, 7/72 infected, respectively), and a higher proportion of birds sampled in July were infected compared to birds sampled in June (prevalence = 12.5%, 11/88 infected; 6.25%, 1/16 infected, respectively) (Fig. 2). We did not observe infected birds at two of our sampling locations (RAMP and SACR). In contrast, over 15% of birds at NESU and FLTO were infected (Fig. 2).

**Fig. 3:**
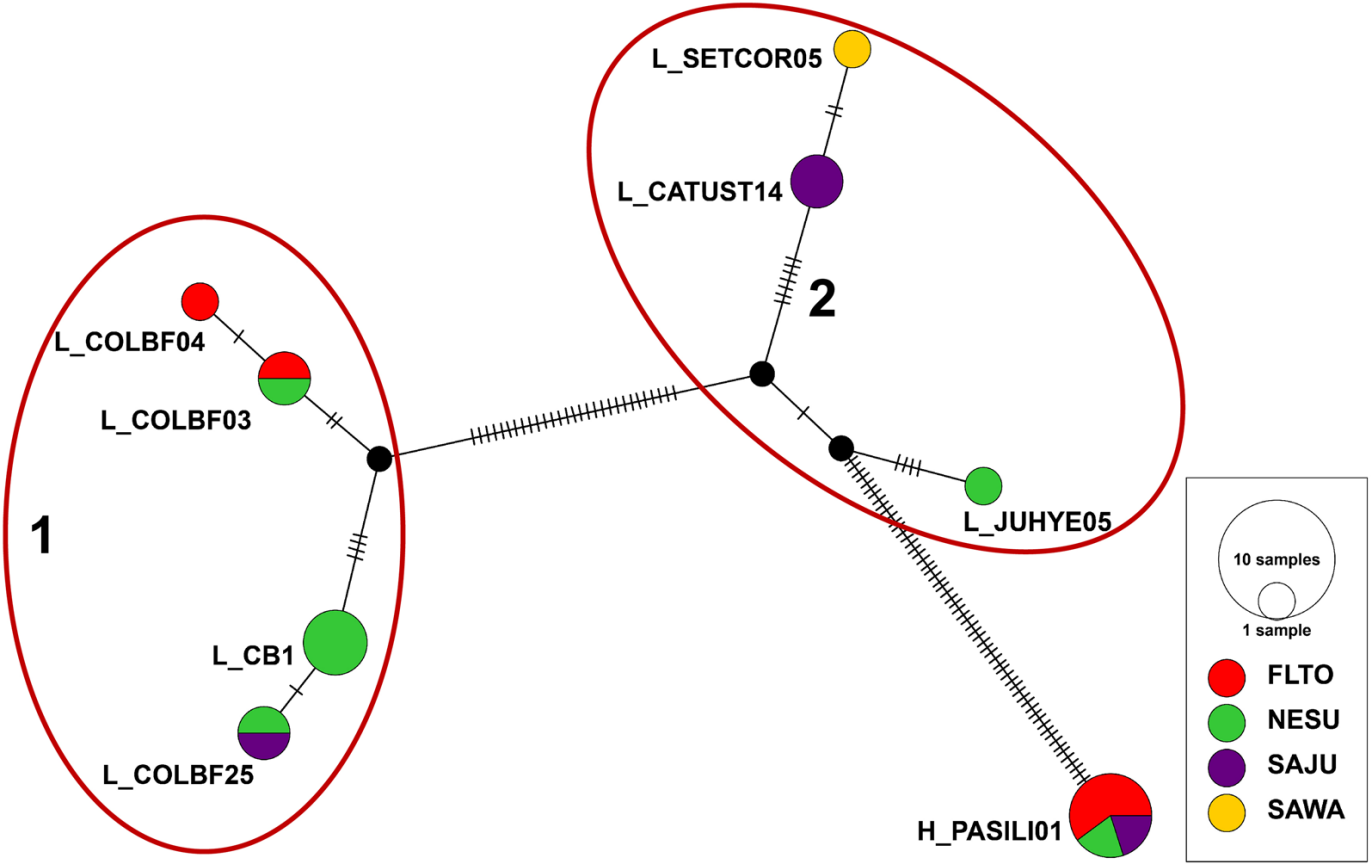
Network plot shows haemosporidian lineages by sampling site (indicated by color). Each hatch mark indicates a single base pair difference between lineages, and black circles show missing lineages. The *Haemoproteus* lineage “H_PASILI01” is observably divergent from *Leucocytozoon* lineages (designated with an “L”). *Leucocytozoon* lineages form two major clusters. All lineages in Cluster 1 have the same insect vector, *Simulium silvestre*. This plot was generated using the program PopArt [20]

We identified eight different haemosporidian lineages that aligned 100% with lineages previously reported to the MalAvi database. Seven belonged to the genus *Leucocytozoon*, and a single lineage was in the genus *Haemoproteus.* All lineages beginning with an “L” are associated with *Leucocytozoon*. Similarly, “H” is used to distinguish the single *Haemoproteus* lineage (Table 2, Fig. 2). Our Median-Joining network analysis shows the *Haemoproteus* lineage H_PASILI01 is clearly diverged from all *Leucocytozoon* lineages (Fig. 3). Reticulation events based on *cytb* data show that *Leucocytozoon* lineages appear to cluster into two groups that we refer to as Clusters 1 and 2 (Fig. 3). Based on data reported to the MalAvi database (retrieved September 2022, summarized in Table 2), all lineages in Cluster 1 have previously been reported in Colorado and are vectored by the same black fly species, *Simulium silvestre*. None of the lineages in Cluster 2 have a previously reported vector but all have been described infecting birds in Western North America.

**Table 2:** Haemosporidian infections detected in Brown-capped Rosy-Finches (*Leucosticte australis*) sampled in 2018

Lineage^a^	Known range^a^	Known host families^a^	Known vector(s)^a^	Sites where detected
L_CATUST14	Western North America	Turdidae, Parulidae, Motacillidae, Paridae, Sylviidae, Fringillidae, Trochilidae	N/A	SAJU
L_CB1	Widespread in North America with few reports in Europe and Asia	Fringillidae, Paridae, Parulidae, Sylviidae, Certhiidae, Motacillidae, Corvidae, Gruidae, Hirundinidae	*Simulium silvestre*	NESU
L_COLBF03	Colorado Rocky Mountains	N/A	*Simulium silvestre*	NESU, FLTO
L_COLBF04	Colorado Rocky Mountains	N/A	*Simulium silvestre*	FLTO
L_COLBF25	New Mexico Sky Islands	Parulidae	*Simulium silvestre*	SAJU, NESU
L_JUHYE05	Alaska	Fringillidae	N/A	NESU
L_SETCOR05	Alberta	Parulidae	N/A	SAWA
H_PASILI01	Western North America	Fingillidae, Parulidae, Corvidae	N/A	SAJU, NESU, FLTO

^a^Based on information obtained from the MalAvi database [13]

## Discussion

The extent to which parasite infections might influence Brown-capped Rosy-Finch survival and reproductive success is unknown. However, we report that these high elevation specialists do indeed experience infections with haemosporidian parasites. Based on molecular identification we found that 11.5% (12/104) of our sampling population was infected. Given variation in the success of primers and the potential for birds to harbor very low infection loads [23, 24], our reported prevalence data should be considered minimum estimates. We did not statistically compare prevalence based on sex, sampling month, and site because of the small sample size.

We did not observe infections with *Plasmodium* parasites, likely because insect vectors associated with *Plasmodium* are more common at lower elevations as has been suggested in other studies [25, 26]. *Plasmodium* parasites are known to be transmitted by mosquitoes (Diptera: Culicidae) typically in the genera *Culex*, *Culiseta*, and *Aedes* [1]. Surveys including a common vector for avian *Plasmodium* parasites, *Culex pipiens*, have reported that the vector does not occur above 1700 m in elevation along the Colorado Front Range [27, 28]. In contrast, black flies (Diptera: Simuliidae) are the known vector for *Leucocytozoon* parasites. We might expect these infections to be common at high elevations, where Brown-capped Rosy-Finches occur, given that vector species such as *Simulium silvestre* are known to breed in small fast-flowing streams that are common in the alpine area [29]. In songbirds, *Haemoproteus* infections are reportedly vectored by biting midges of the genus *Culicoides* [1, 30]. There is a lack of literature on the diversity and distribution of *Culicoides* in Colorado, but, in general, these insects are known to breed in moist areas with high levels of organic material such as ponds [31]. Of the sites where we report *Haemoproteus* infections, all were in close proximity to bodies of water.

We observed higher infection prevalence in female Brown-capped Rosy-Finches compared to males. Although the underlying cause of this disparity could be our sampling size, further research on Brown-capped Rosy-Finch breeding behavior and physiology might provide insight. Infections were more common in July than in June. While this might be due to sampling sites that were visited in June having inherently fewer infected birds (SAWA, RAMP, SAJU, and SACR), this difference could also be due to infections manifesting later in the summer because of both host physiology and vector phenology. Conversely, prevalence differences that we report by site could also be influenced by the timing of when birds were sampled. Testing samples collected from birds at the same field sites spanning multiple time points would resolve this knowledge gap. Additionally, climate varies across the Colorado Rocky Mountains, and this can influence the presence of vectors. For example, based on high-resolution climate mapping from the PRISM climate group [32], between 1981–2010, RAMP, a site with prevalence = 0%, exhibited an average annual rainfall < 90 cm. We also note that NESU, our most northerly site and the second lowest in elevation, had the highest prevalence of 22%. Both precipitation and elevation potentially influence Brown-capped Rosy-Finch exposure to vectors and subsequent haemosporidian infections.

From searching the MalAvi database, both lineages L_CB1 and L_CATUST14 appear to be host generalists, infecting multiple avian families (Table 2) [13]. L_CB1 is widespread throughout North America whereas reports of L_CATUST14 are more restricted to Western North America. Other *Leucocytozoon* lineages also appear more restricted in range, with two lineages, L_COLBF03 and L_COLBF04, having only previously been reported to the MalAvi database infecting the insect vector *Simulium silvestre* in the Colorado Rocky Mountains. However, infections with these two lineages have been observed in 12-day-old Black-capped Chickadees (*Poecile atricapillus*) and Mountain Chickadee (*Poecile gambeli*) nestlings in Boulder County, Colorado [33]. All lineages in Cluster 1 have been associated with the same insect vector, *Simulium silvestre*, in a previous study conducted in the Colorado Rocky Mountains [34].

Results from our Median-Joining network analysis, combined with previous reports made to the Malavi database, show that four of the closely related lineages that infect Brown-capped Rosy-Finches (L_CB1, L_COLBF03, L_COLBF04, and L_COLBF25, i.e. Cluster 1), are all vectored by the same black fly species: *Simulium silvestre*. The distribution and abundance of this vector might therefore be an effective predictor of infections with multiple *Leucocytozoon* lineages in Brown-capped Rosy-Finches. However, it is possible that these same lineages are transmitted by additional vectors that have not yet been described. Vectors for all other haemosporidian lineages we observed infecting Brown-capped Rosy-Finches have not yet been reported to the MalAvi database.

## Conclusions

Here we reported on a survey of Brown-capped Rosy-Finch haemosporidian parasites (Genera: *Plasmodium*, *Haemoproteus*, and *Leucocytozoon*). To our knowledge, no previous literature has identified *Leucocytozoon* or *Haemoproteus* infections in this high alpine specialist, and we documented both genera at relatively low prevalence. Half of the haemosporidian lineages we documented (4/8) were reported in the same insect vector, *Simulium silvestre*, in a previous study [34]. We did not examine blood smears using microscopy methods, which prevents us from being able to quantify infection intensities and verify that the reported haemosporidian parasites are completing their life cycles in Brown-capped Rosy-Finches. However, our report provides a baseline understanding of the blood parasite infections that this endangered high elevation specialist harbors. With ongoing climate warming we might expect the haemosporidian communities infecting Brown-capped Rosy-Finches to shift over time, given potential movement of vector ranges.

## Supplementary Information

**Additional file 1: Dataset S1.** Brown-capped Rosy-Finch capture data show individual birds by their USGS band number. For each bird, information on their sex, sampling location, the elevation of sampling (in meters), year and month of capture, infection status, and haemosporidian lineage(s) (if applicable).

## Data Availability

Blood samples for this work are available at the Denver Museum of Nature and Science. See Additional file 1: Table S1 for Brown-capped Rosy-Finch infection status data. Haemosporidian lineages identified from this work are described on the MalAvi database.
